# Antimicrobial Effect of Isotretinoin Therapy on Periodontal Pathogens: A Case-Control Study

**DOI:** 10.3390/antibiotics10111286

**Published:** 2021-10-21

**Authors:** Reham AlJasser, Razan AlAqeely, Afnan AlZahrani, Manal AlKenani, Sadeem AlQahtani, Mohammed AlSarhan, Dalal AlOtaibi, Rhodanne Lambarte

**Affiliations:** 1 Department of Periodontics and Community Dentistry, College of Dentistry, King Saud University, Riyadh 11545, Saudi Arabia; raqeely@ksu.edu.sa (R.A.); malsarhan@ksu.edu.sa (M.A.); dalalotaibi@ksu.edu.sa (D.A.); 2 Ministry of Health, Riyadh 11176, Saudi Arabia; afnan36x@gmail.com; 3 Saudi Board of Periodontics, Riyadh 11545, Saudi Arabia; manalkenani95@gmail.com (M.A.); sadeemsq15@gmail.com (S.A.); 4 Molecular and Cell Biology Laboratory, Prince Naif Bin AbdulAziz Health Research Center, College of Dentistry, King Saud University, Riyadh 11545, Saudi Arabia; rlambarte@ksu.edu.sa

**Keywords:** oral microbes, periodontal pathogens, isotretinoin, periodontal therapy

## Abstract

Isotretinoin (INN), a drug used to treat severe acne, has anti-inflammatory and antibacterial properties. INN may affect periodontal pathogenic bacteria, so we aimed to study the effect of INN on intraoral microbial profiles of periodontal disease and healthy periodontium. Our case-control study divided 180 subjects into six groups according to periodontal health status and INN usage as follows: healthy periodontium receiving INN (HINN; *n* = 30); those with generalized plaque-induced gingivitis receiving INN (GINN; *n* = 30); and those with stage I generalized periodontitis receiving INN (PINN; *n* = 30). Subjects not taking INN, were categorized in the same manner: those with a healthy periodontium (HC; *n* = 30); those with generalized plaque-induced gingivitis (GC; *n* = 30); and those with generalized periodontitis stage I (PC; *n* = 30). Plaque samples were collected to determine the prevalence of four periodontal pathogens (*Porphyromonas gingivalis*, *Tannerella forsythia*, *Treponema denticola*, and *Fusobacterium nucleatum)* in each study group using real-time polymerase chain reaction. Data were analyzed using IBM SPSS software, and multiple regression analysis was performed for each parameter tested in each group at a significance level of 0.05. All INN groups showed significantly lower levels of *P. gingivalis*, *T. forsythia*, and *T. denticola* and higher levels of *F. nucleatum* (*p <* 0.001). INN had an observable antimicrobial effect on the periodontal pathogen count in patients with plaque-induced gingivitis and chronic periodontitis. INN may have a potential additive antimicrobial value in the treatment of periodontal disease.

## 1. Introduction

Periodontitis is a multifactorial biofilm-induced disease that causes attachment loss and alveolar bone resorption [1]. Several studies have shown that areas in the oral cavity are inhabited by a wide variety of microbes in the form of biofilms, which can alter periodontal health and disease status [2,3]. In periodontally healthy sites, the microbial biofilm primarily consists of Gram-positive facultative species and members of *Streptococci* and *Actinomyces*, as well as small amounts of Gram-negative bacteria [4]. The shift in microbial biofilms associated with plaque-induced gingivitis consists of roughly equal proportions of aerobic (Gram-positive and Gram-negative species), facultative, and anaerobic microorganisms. Most of these species also populate in large numbers in established periodontitis.

Gram-positive bacteria most associated with gingivitis are *Streptococcus* species, and Gram-negative bacteria are predominantly *Fusobacterium* species. The most common pathogens in periodontitis are *Porphyromonas gingivalis* and *Tannerella forsythia* [5,6].

Dysbiosis in the periodontal environment leads to further breakdown of periodontal tissue. However, resolving tissue inflammation and periodontal destruction is possible by altering the plaque biofilm with mechanical and chemotherapeutic methods [7].

Systemic changes can alter the body’s ability to adapt, even with proper plaque control, and can shift to a dysbiotic bacterial community. Physiological factors, including age and hormonal changes (e.g., puberty, pregnancy, and menopause), may also contribute to this type of shift [8].

Another manifestation of hormonal imbalance is acne. Androgens increase sebum production, which leads to pore clogging, creating a habitable environment for *Propionibacterium acnes* [9]. The inflammatory consequences of acne, especially in severe, chronic cases, often cause people to seek treatment, in addition to its psychological and social effects [10]. Several therapies were recommended; they range from topical treatments such as benzoyl peroxide, retinoids, and antibiotics to orally administered treatments such as antibiotics, hormones, and oral isotretinoin in moderate and severe cases [11].

Isotretinoin (INN) is a vitamin A active metabolite that is one of the most widely used drugs to treat severe acne. It is an orally administered retinoid that targets the etiological factors of acne [12]. This medication has antibacterial and anti-inflammatory properties, as it appears to reduce *P. acnes* and *Staphylococcus epidermidis* in facial sebum [13]. INN has shown effectiveness in clearance of disease and controlling inflammatory acne and hyperseborrhea compared to systemic antibiotics [11]. Its side effects varied from cutaneous and extracutaneous side effects to teratogenicity; however, adherence to the regimen and closed monitoring by specialists is paramount [11,14]. The recommended dosage according to European guidelines are 0.3–0.5 mg/kg/day for nodular/papulopustular acne [15].

Recently, some paper correlated the effect of INN on different periodontal disease status. Clinical and biological signs of inflammation were found in a group of the population using INN medication and having active periodontal disease as MMP-8 and MMP-9 salivary levels as well as TIMP-1 and -2 levels [16,17].

In the present study, we aimed to evaluate changes in the microbiological profile, specifically with red complex bacteria, which causes periodontal disease, of patients receiving at least three months of oral INN treatment for cutaneous acne, compared with patients with gingival and periodontal diseases and those with a healthy periodontium.

## 2. Results

Samples were taken from total of 180 participants, where 44% were male and 56% were females. Age mean and standard deviation were as follow for subjects including HC, GC, PC with values of 24.9 (5.4), 25.2 (7.8), and 26.2 (2.8), respectively. INN groups HINN, GINN, and PINN with values of 24.7 (3.5), 23.6 (3.8), and 24.1 (3), respectively. Intra- and interexaminer reliability were good according to Kappa statistics.

**Figure 1:**
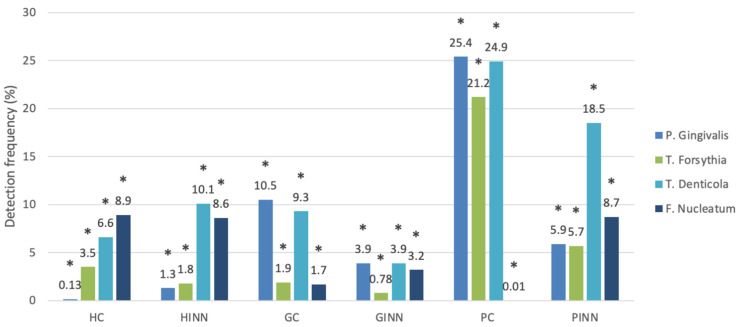
Bacterial detection frequencies of the four periodontal pathogens among the six study groups determined by real-time PCR. HC, healthy periodontium; HINN, subjects with healthy periodontium receiving isotretinoin (INN); GC, control subjects with gingivitis; GINN, generalized plaque-induced gingivitis; PC, control subjects with generalized periodontitis stage I; PINN, subjects with generalized periodontitis stage I receiving INN; *, statistically significant.

The detection frequency of periodontal pathogens determined by r-PCR is shown in Figure 1. In general, *P. gingivalis* levels were lower in the healthy periodontium (HC) (0.13%) and subjects with healthy periodontium receiving INN (HINN; 1.3%) groups than in the other four groups (generalized plaque-induced gingivitis (GINN), control subjects with gingivitis (GC), subjects with generalized periodontitis stage I receiving INN (PINN), and control subjects with generalized periodontitis stage I (PC)). Among these four groups, *P. gingivalis* levels were higher in the PC group (25.4%), followed by the GC, PINN, and GINN groups (10.5%, 5.9%, and 3.9%, respectively). These differences were statistically significant (*p* < 0.001)(Table 1).

**Figure 2:**
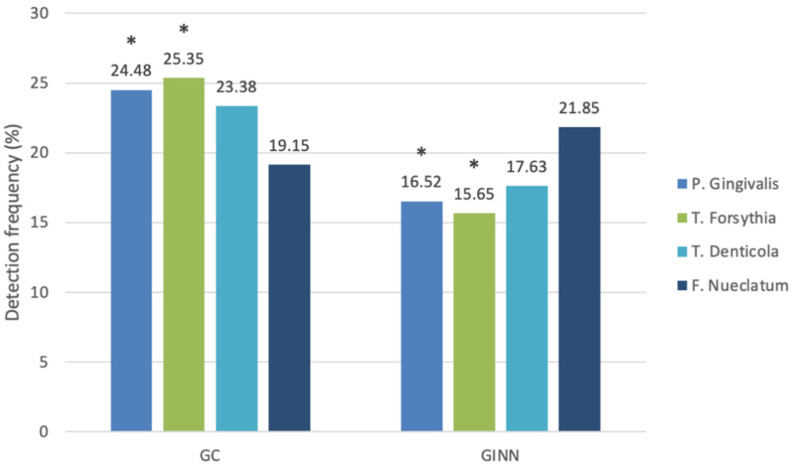
Comparison between control subjects with gingivitis (GC) and subjects with gingivitis receiving isotretinoin (INN) (GINN) in the detection of bacterial pathogens. *, statistically significant.

Another significant finding was in the *T. forsythia* levels, which were lower in the GINN (0.78%), HINN (1.8%), and GC (1.9%) groups than in the other three groups: HC (3.5%), PINN (5.7%), and PC (21.2%). The comparison of *Treponema denticola* values was statistically significant, showing lower levels in the GINN (3.9%), HC (6.6%), and GC (9.3%) groups compared with the other three groups: HINN (10.1%), PINN (18.5%), and PC (24.9%). However, *Fusobacterium nucleatum* levels were significantly lower in the PC, GC, and GINN groups (0.01%, 1.7%, and 3.2%, respectively) than in the other three groups (HINN, HC, and PINN) (Table 2 and Table 3 and Figure 1 and Figure 2).

Among the gingivitis groups (Table 2 and Figure 2), *P. gingivalis* and *T. forsythia* levels were significantly higher in the GC group (24.48% and 25.35%, respectively) than those in the GINN group (*p =* 0.032 and 0.009, respectively). Conversely, there were no statistically significant differences in *T. denticola* and *F. nucleatum* levels between the GINN and GC groups (*p =* 0.12 and 0.465, respectively).

**Figure 3:**
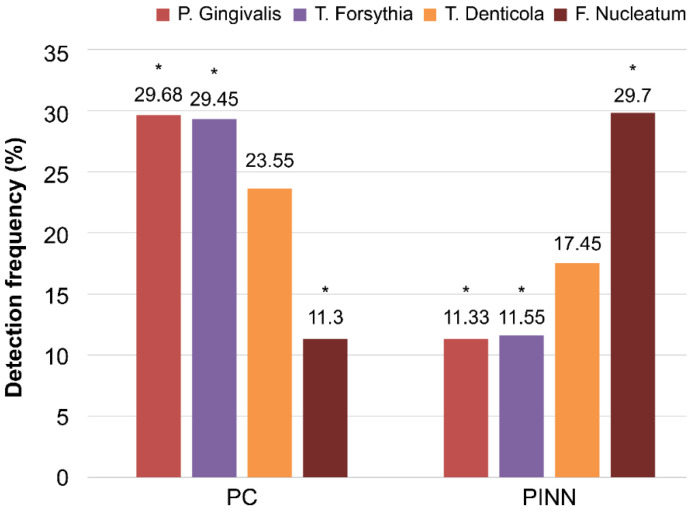
Comparison between control subjects with generalized periodontitis stage I (PC) and subjects with generalized periodontitis stage I receiving isotretinoin (INN) (PINN) in the detection of bacterial pathogens. * statistically significant.

*P. gingivalis* and *T. forsythia* levels were significantly higher in the PC group (29.68% and 29.45%, respectively) than in the PINN group. Furthermore, *F. nucleatum* levels in the PINN group were significantly higher (29.70%) than those in the PC group (11.30%). However, there were no significant differences in *T. denticola* levels between the PINN and PC groups (*p =* 0.098) (Table 4 and Figure 3).

## 3. Discussion

Various oral microorganisms are found in dental plaque, with an average bacterial count ranging between 12 and 27 species in a small sample [2,18]. Under certain conditions, changes in the composition and properties of dental plaque biofilms can lead to oral conditions such as periodontal diseases [19]. Several studies have linked the progression of periodontal disease to the combined action of pathogens [20,21]. Oral isotretinoin has been found to have an antibacterial effect on facial sebum [13]. However, no studies have examined the impact of isotretinoin on periodontal pathogens in the oral cavity, but rather examined salivary flow rate and tissue biomarkers. It was concluded that isotretinoin has reduced salivary flow and salivary MMPs, specifically 8 and 9, as well as elevated salivary TIMP-1 and TIMP-2 [16,17]. In this study, we determined the changes in oral bacteria during systemic isotretinoin administration using plaque samples and real-time PCR detection.

The present study illustrated the relationship between the periodontal microbial profile and the use of isotretinoin. All groups taking INN with different periodontal diagnoses showed significantly lower *P. gingivalis*, *T. forsythia*, and *T. denticola* levels in the dental plaque. These microbiomes are defined as red complexes and are well-known anaerobic pathogens strongly linked to the onset and progression of periodontal disease [22]. On the contrary, the same study groups showed significantly higher levels of *F. nucleatum*, which are correlated with periodontal health status [23]. Differences were more pronounced clinically and statistically among patients diagnosed with stage I generalized periodontitis. Therefore, these results confirm the antimicrobial properties of this medication. Although the mechanism of action is unknown, several reports have supported the antibacterial effect of isotretinoin, specifically on Gram-negative and anaerobic microorganisms such as *P. acnes* [24]. Leyden et al. (1986) reported qualitative and quantitative changes in skin bacteria during isotretinoin therapy, resulting in a marked reduction in *P. acnes* and Gram-negative bacteria, and an increase in *Staphylococcus aureus* [25]. King et al. (1982) reported a significant reduction in microbial counts with the use of isotretinoin. However, the effect was secondary, caused by changes in sebum excretion. The reduction was more significant in microorganisms found in lipid-rich regions, such as *Propionibacteria* and *Pityrospora* [26].

In terms of intraoral investigations, only two studies explored the cariogenic effect of this medication, and both reported no significant impact on intraoral cariogenic pathogen counts in patients treated with INN [27,28]. This study could not address this effect according to the current study design.

This study has a potential limitation, as the data were obtained only once, without further follow-up. Monitoring the patients periodontally during the INN course regime might result in different responses. Additionally, this might help in monitoring oral side effects of INN. Considering that the medication is used for a prolonged period, varying INN responses are possible among individuals. However, it should be noted that this is considered the first study to evaluate the antimicrobial effect of this medication from a dental and periodontal perspective. Further longitudinal microbiological analysis of periodontal pathogens during the INN course and after shifting to topical INN could be of interest and importance.

## 4. Materials and Methods

### 4.1. Study Sample

The sample size of 180 subjects was determined using G Power software. The confidence level was set at 95%, and a power level of 80% was defined with a moderate effect size. There were 30 subjects in each of six study groups [29].

Clinical measurements were performed on two randomly selected subjects by the two examiners (A.Z. and M.K.). These measurements have been repeated after 10 days and Cohen’s kappa score was used to measure reliability level.

### 4.2. Subjects

Plaque samples were obtained from patients 18 years and older. The subjects who participated in this study were patients from the Department of Dermatology of King Khalid University Hospital. The study protocol (E-19-3856) was approved by the Institutional Review Board (IRB) of King Saud University Medical City. Written informed consent was obtained from all subjects involved in the study.

All subjects who received a 0.5 or 1.0 mg/kg/day dose of INN (Roaccutane^®^) and did not receive periodontal treatment or antibiotic therapy three months before the investigation were included. After the periodontal examinations were performed at the Dental University Hospital at King Saud University, the patients were divided into the following three groups: those with a healthy periodontium receiving INN (HINN; *n* = 30); those with generalized plaque-induced gingivitis receiving INN (GINN; *n* = 30); and those with stage I generalized periodontitis receiving INN (PINN; *n* = 30). Negative control groups, comprised of subjects not taking INN, were categorized in the same manner: those with a healthy periodontium (HC; *n* = 30); those with generalized plaque-induced gingivitis (GC; *n* = 30); and those with generalized periodontitis stage I (PC; *n* = 30) [16,30]. Exclusion criteria included the long-term use of medications that affect salivary flow or periodontal status, a history of autoimmune diseases, metabolic bone diseases, diabetes, or postmenopausal osteoporosis. Pregnant women and smokers were also excluded from the study.

### 4.3. Microbiological Sample Collection and Preparation

An examiner collected plaque samples to standardize the sampling procedure. Before sampling, the selected tooth and adjacent teeth on each side were isolated using cotton rolls. A sterilized universal curette was used for sampling to collect the accumulated plaque around the right and left lower first molars in subjects who did not have a deep periodontal pocket. In subjects with periodontal pockets (≥4 mm), plaque samples were collected from the deepest pocket. The collected plaque was then placed in phosphate-buffered saline (0.5 mL) in sterilized 1.5 mL Eppendorf tubes and stored at −80 °C until further analysis. Microbial DNA was isolated and pooled from paper points using a PureLink microbiome DNA purification kit, and DNA concentrations were quantified using a Qubit 4 fluorimeter and dsDNA BR assay kits (Exgene™ Cell SV, GeneAll^®^ Biotechnology, Seoul, Korea). The samples were maintained at The Eppendorf BioSpectrometer^®^ basic (Hamburg, Germany) was used to evaluate DNA quality and measure relative quantity [31].

### 4.4. Q-PCR Analysis

Quantitative real-time polymerase chain reaction (q-PCR) was used to detect *P. gingivalis*, *T. forsythia*, *T. denticola*, and *F. nucleatum* in the collected plaque samples. 5× HOT FIREPol^®^ EvaGreen^®^ qPCR Supermix (Solis BioDyne, Tartu, Estonia) was used to amplify bacterial DNA. Specific primers were used for each proposed bacterium and a universal primer (16S rRNA) for standard bacteria to verify the presence of bacterial DNA and allow relative quantification. Table 1 lists the sequences of the primers obtained from Macrogen, Inc. (Seoul, Korea) (Table 1).

### 4.5. Data Analysis

A statistical analysis was performed using SPSS 21.0 version software (IBM Inc., Chicago, IL, USA). The study and outcome variables were described using descriptive statistics (mean, standard deviation, median, interquartile range, frequencies, and percentages). Nonparametric statistical tests (Kruskal–Wallis test and Mann–Whitney *U*-test) were used to compare the mean ranks of the outcome variables in relation to six study groups and between two groups since the outcome variables were skewed. The distribution of categorical responses was tested using the Pearson chi-square test. Statistical significance was set at a *p*-value ≤ 0.05.

## 5. Conclusions

The results indicate an antimicrobial effect of INN on periodontal parameters in patients with plaque-induced gingivitis and periodontitis, with potential applications in periodontal treatment. INN groups showed significantly lower levels of *P. gingivalis*, *T. forsythia*, and *T. denticola* and higher levels of *F. nucleatum*. INN had an observable antimicrobial effect on the periodontal pathogen count in patients with plaque-induced gingivitis and chronic periodontitis. Future well-designed longitudinal and clinical trials are recommended to validate and further investigate these findings.

## Figures and Tables

**Table 1:** Sequence of primers used in quantitative real-time polymerase chain reaction.

Species	Sequence (5′-3′)	Product Size (16S rRNA)
*Porphyromonas gingivalis*		
Forward primer	5′-TAC CCA TCG CCT TGG T-3′	19 bp
Reverse primer	5′-CGG ACT AAA ACC GCA TAC ACT TG-3′	23 bp
*Fusobacterium nucleatum*		
Forward primer	5′-CGC AGA AGG TGA AAG TCC TGT AT-3′	23 bp
Reverse primer	5′-TGG TCC TCA CTG ATT CAC ACA GA-3′	23 bp
*Tannerella forsythia*		
Forward primer	5′-ATC CTG GCT CAG GAT GAA CG-3′	20 bp
Reverse primer	5′-TAC GCA TAC CCA TCC GCA A-3′	19 bp
*Treponema denticola*		
Forward primer	5′-AGA GCA AGC TCT CCC TTA CCG T-3′	22 bp
Reverse primer	5′-TAA GGG CGG CTT GAA ATA ATG A-3′	22 bp

**Table 2:** Comparative statistics of outcome variables across the six study groups.

Variables	HC	HINN	GC	GINN	PC	PINN	*p*-Value
Polymerase chain reaction (%)							
*Porphyromonas gingivalis* Mean (SD)	0.13 (0.22)	1.3 (2.4)	10.5 (12.9)	3.9 (6.4)	25.4 (11.0)	5.9 (5.9)	<0.001 *
*Tannerella forsythia* Mean (SD)	3.5 (6.2)	1.8 (4.5)	1.9 (2.1)	0.78 (1.2)	21.2 (9.3)	5.7 (5.5)	<0.001 *
*Treponema denticola* Mean (SD)	6.6 (9.6)	10.1 (9.3)	9.3 (17.9)	3.9 (9.3)	24.9 (10.7)	18.5 (11.5)	<0.001 *
*Fusobacterium nucleatum* Mean (SD)	8.9 (15.9)	8.6 (6.7)	1.7 (3.7)	3.2 (5.6)	0.01 (0.02)	8.7 (16.2)	<0.001 *

*, statistically significant; HC, control subjects with healthy periodontium; HINN, subjects with healthy periodontium receiving isotretinoin (INN); GC, control subjects with gingivitis; GINN, subjects with gingivitis receiving INN; PC, control subjects with generalized periodontitis stage I; PINN, subjects with generalized periodontitis stage I receiving INN; SD, standard deviation.

**Table 3:** Comparison of mean ranks of outcome variables between the gingivitis groups.

Variables	GINN		GC		*p*-Value
	Median	Mean	Median	Mean	
	(IQR)	Ranks	(IQR)	Ranks	
Polymerase chain reaction (%)					
*Porphyromonas gingivalis*	1 (4.6)	16.52	3.6 (22.4)	24.48	0.032 *
*Tannerella forsythia*	0.3 (0.6)	15.65	1.3 (1.9)	25.35	0.009 *
*Treponema denticola*	0.8 (3.6)	17.63	1.1 (9.9)	23.38	0.12
*Fusobacterium nucleatum*	0.8 (2.2)	21.85	0.5 (1.4)	19.15	0.465

*, statistically significant; GC, control subjects with gingivitis; GINN, subjects with gingivitis receiving isotretinoin (INN); IQR, interquartile range.

**Table 4:** Comparison of mean ranks of outcome variables between the periodontitis groups.

Variables	PC		PINN		*p*-Value
	Mean Ranks	Median	Mean Ranks	Median	
		(IQR)		(IQR)	
Polymerase chain reaction (%)					
*Porphyromonas gingivalis*	29.68	23.1 (17.9)	11.33	3.8 (8.2)	<0.0001 *
*Tannerella forsythia*	29.45	18.8 (7)	11.55	3.2 (7.6)	<0.0001 *
*Treponema denticola*	23.55	23.1 (15.8)	17.45	18.2 (16.6)	0.098
*Fusobacterium nucleatum*	11.3	0.01 (0.03)	29.7	2.3 (2.9)	<0.0001 *

*, statistically significant; PC, control subjects with generalized periodontitis stage I; PINN, subjects with generalized periodontitis stage I receiving isotretinoin (INN); IQR, interquartile range.

## Data Availability

All data are presented in the paper.
